# The impact of sleep quality on visual working memory varied with the duration of maintenance

**DOI:** 10.3389/fpsyg.2024.1404989

**Published:** 2024-06-21

**Authors:** Li Gong, Mengwei Wang, Chaoxiong Ye, Qiang Liu

**Affiliations:** ^1^Institute of Brain and Psychological Sciences, Sichuan Normal University, Chengdu, China; ^2^School of Education, Anyang Normal University, Anyang, China; ^3^Department of Psychology, University of Jyvaskyla, Jyväskylä, Finland

**Keywords:** sleep quality, visual working memory, maintenance, recall task, processing stage

## Abstract

**Objective:**

Sleep quality can affect the performance of visual working memory. However, the effect of sleep quality on the maintenance stage, which is the key to maintain the quality and efficiency of visual working memory representation, remains unclear. This study is the first to explore the effect of sleep quality on the maintenance of visual working memory information.

**Method:**

60 healthy college students completed the Pittsburgh Sleep Quality Index (PSQI) and color recall task of visual working memory. A mixed experimental design of sleep quality (high or low) and delay duration (1, 4, or 6 s) was used to assess the effect of sleep quality on the maintenance phase of visual working memory.

**Results:**

The main effects of sleep quality were significant on visual working memory quantity, precision and offset indexes. Among the quantity index, the interaction between sleep quality and delay duration was also significant. This suggests that prolonging the delay time in the maintenance phase leads to difficulty in maintaining attention to the task for those with lower sleep quality, which results in poorer working memory quantitative representations.

**Conclusion:**

Increases in the delay duration of the maintenance phase in visual working memory intensify the impact of sleep quality on task performance. Our study provides evidence to reveal the relationship between sleep quality and visual working memory and offers recommendations for improving sleep quality and cognitive functioning in individuals.

## 1 Introduction

Numerous studies have found that sleep is particularly crucial for human memory (Walker, 2010; Girardeau and Lopes, 2021; Stanyer et al., 2022; Teräs et al., 2023). As an essential physiological process for an organism’s survival, sleep provides our brains with a time window devoid of a substantial information load, allowing for the classification, organization, and consolidation of newly encoded memories (Klinzing et al., 2019; Brodt et al., 2023). Consequently, compromised sleep quality in an individual can have a significant impact on the memory system.

Visual working memory is a short-term memory system that is specifically responsible for the temporary processing, storage, and manipulation of visual information (Baddeley and Hitch, 1974), and it is crucial for individual learning and development (Dubuc et al., 2020; Verschooren et al., 2021). Previous studies have found that an individual’s sleep status can predict performance in tasks involving visual working memory as a core cognitive function (MacDonald et al., 2018; Xie et al., 2019; Almarzouki et al., 2022). For example, MacDonald et al. (2018) used a recall paradigm to investigate the effects of napping on visual working memory. The experimental results showed that napping significantly improved both the quantity and precision of an individual’s visual working memory. Xie et al. (2019) also employed the visual working memory recall paradigm to investigate the influence of an individual’s daily sleep quality on visual working memory representation. They utilized the Pittsburgh Sleep Quality Index (PSQI) to assess each participant’s daily sleep quality, and they administered the Patient Health Questionnaire-9 (PHQ-9) to control for the impact of emotional variables on visual working memory. Their research findings demonstrated that even after controlling for the influence of emotional variables, sleep quality continued to predict the quantity of visual working memory.

However, previous studies examined only the relationship between sleep quality and visual working memory performance, without delving into the potential mechanisms underlying this relationship. An important consideration is that visual working memory is a complex cognitive system that involves multiple cognitive processes and synergy between multiple brain regions (Delgado Reyes et al., 2020). For visual working memory processing, once the visual stimuli are encoded and consolidated into visual working memory, they enter the memory maintenance phase. The maintenance phase is a crucial stage in the memory process, as it involves the retention and preservation of stored information for subsequent processing and retrieval, supported by multiple neural mechanisms (Kim, 2019; Quentin et al., 2019; Tokuhara et al., 2021). During this maintenance period, individuals need to keep their attention focused on the memorized information and resist the influence of interfering factors. In summary, memory information that enters the maintenance phase exhibits high stability and accessibility. These characteristics are critically important for maintaining the quality and efficiency of working memory representations (Stickgold and Walker, 2007; Heinrichs-Graham and Wilson, 2015).

Earlier studies have found that insufficient sleep accelerates individual decay on continuously presented auditory and visual items (Raidy and Scharff, 2005; Liberalesso et al., 2012). This means that poor sleep causes damage to perceptual processes, which in turn affects an individual’s ability to maintain sensory and perceptual information (Li et al., 2024). Specifically, sleep-deprived individuals are in an unstable state, leading to reduced information accumulation and increased variability, which affects information maintenance. Furthermore, a large number of studies have shown that poor sleep quality or sleep deprivation reduces neural activity in relevant brain regions (such as the prefrontal and parietal lobes, etc.), leading to changes in behavioral performance (Muzur et al., 2002; Herrero Babiloni et al., 2021). And these brain regions are closely related to the maintenance phase of visual working memory (Kim, 2019; Sahu and Tseng, 2021). On the other hand, from the perspective of neuroplasticity, the synaptic connections between neurons are the basis of brain adaptation and learning, and it also plays an important role in the formation and maintenance of visual working memory (Jeong et al., 2021; Yao et al., 2023). Whereas, insufficient sleep impairs synaptic connections between neurons, which affect the storage and maintenance of visual working memory (Klinzing et al., 2019). All of the above provide indirect evidence that sleep quality affects the maintenance of visual working memory information.

In addition, the increase in delay time in the visual working memory maintenance phase may be accompanied by a continuous accumulation of error information (Panichello et al., 2019). This means that the longer the delay time, the weaker the individual’s ability to maintain memorized information. For example, Masse et al. (2019) found that short-term synaptic plasticity can support information maintenance within sufficiently short memory delay periods. Conversely, if the delay period is prolonged, the maintenance of working memory information will be impaired. Therefore, to simply investigate the ability of individuals to maintain visual working memory information from a behavioral perspective, it can be achieved by manipulating the delay time of the maintenance phase.

In the present study, it is reasonable to assume that the impact of individual sleep quality on visual working memory is primarily based on the effect on the ability to maintain information. Unfortunately, research that directly addresses and validates this issue remains scarce. This study was to combine a sleep quality questionnaire and color recall paradigm to explore the effect of sleep quality on the maintenance of information in visual working memory by varying the delay time of the maintenance phase. The overall goal is to better reveal the essence of how sleep quality affects visual working memory.

## 2 Methods

### 2.1 Participants

All participants were preliminarily screened using the measurement task of a sleep quality questionnaire. The selected sleep quality questionnaire was the Chinese version of the Pittsburgh Sleep Quality Index (PSQI), as revised by Liu and Tang (1996), based on the original version developed by Buysse et al. (1989). The questionnaire consists of 18 items and adopts a 4-point scoring system (0–3 points). The cumulative score of each factor is the total score of the scale. The total score range is 0–21 points, with higher scores indicating poorer sleep quality. According to the clinical detection criteria for sleep quality issues in China, a score of 7 is the detection threshold, and scores ranging from 0 to 5 indicate good sleep quality (Liu and Tang, 1996). A total of 522 (M ± SD = 5.70 ± 2.48) data were collected during the screening phase, and the Cronbach’s alpha coefficient of the scale was 0.80. In this study, we classified participants with scores of 5 or below as the high sleep quality group and those with scores of 8 or above as the low sleep quality group. We then scheduled experiments for those participants who volunteered to participate in further studies.

The experiment was a mixed design of 2 sleep quality levels (high vs. low) × 3 delay durations (1 s vs. 4 s vs. 6 s). The between-subject factor was the ‘sleep quality group’, and the within-subject factor was the ‘delay durations’. An *a priori* power analysis was conducted using G*Power 3.1 to estimate the required sample size for the study. To achieve a significance level of *α* = 0.01, a medium effect size of *f* = 0.25, and an expected statistical power of 80%, a minimum total sample size of 40 was determined. To improve the statistical power of the data, we recruited a total of 63 undergraduate students (n low-scoring group = 32, n high-scoring group = 31) for the experiment based on the initial sleep questionnaire results. Among them, 3 were excluded due to a guessing rate (g) higher than 0.75 in the visual working memory task. Ultimately, 60 participants (n low-scoring group = 30, n high-scoring group = 30) provided valid data. The participants had an average age of 19.23 ± 1.05 years, and 27 (45%) were male. The difference in gender ratio between the two groups of participants was not significant, *χ^2^* = 0.07, *p* = 0.79. All participants had normal or corrected vision without color blindness or color weakness. Participants were given detailed instructions, and informed consent was obtained before the experiment. Rewards were given after the experiment. The study was conducted in accordance with the principles outlined in the Helsinki Declaration, and approval was granted by the Ethics Committee of Liaoning Normal University (Approval number: LL2022032).

### 2.2 Stimuli

The experimental procedure for the visual working memory color recall task was implemented using Matlab 2016b. All stimuli were presented on a 23.8 inch LCD monitor with a refresh rate of 60 Hz and a resolution of 1920 × 1,080 pixels. The screen background was set to gray, with a brightness of 150°. Participants were seated approximately 60 cm away from the display monitor and performed the tasks in a softly lit and quiet room. The presented memory color squares (0.7° × 0.7°) were randomly selected from a uniform distribution of a 360° color wheel (outer diameter of 8.2°, inner diameter of 6°). The color difference between any two squares was greater than 45°. The color squares were presented on an imaginary circle with a radius of 150 pixels centered on a central fixation point (with a radius of 20 pixels). The angular distance between the color squares was 90°.

### 2.3 Procedure

**Figure 1:**
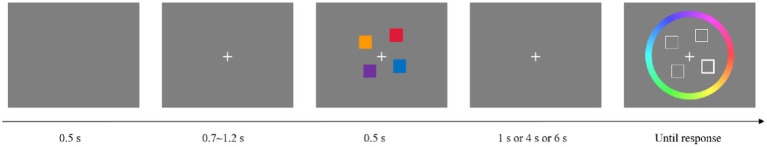
A schematic diagram of the task used in the experiment.

The experimental procedure is illustrated in Figure 1. First, a blank screen was presented for 0.5 s. A fixation point was then displayed for a jittered time window of 0.7 to 1.2 s. Subsequently, a memory array consisting of four color squares was presented for 0.5 s. This was followed by a delay period of 1 s, 4 s, or 6 s (the order was counterbalanced between the participants). Finally, in the response phase, a color wheel and four hollow squares were simultaneously presented. The positions and sizes of the squares matched the four color squares in the memory array. One of the squares was highlighted to indicate the location that required retrieval. The participants used a mouse to respond on the color wheel. The experiment consisted of a total of 300 trials, with 100 trials per condition. Rest periods were provided after every 50 trials, and participants could continue with the experiment by pressing the “Space” key. The entire experiment lasted approximately 40 min.

### 2.4 Statistical analysis

The data from the visual working memory task were analyzed using the Mem Toolbox, and the standard mixture model by Zhang and Luck (2008) was employed, with fitting performed using the standard maximum likelihood method. Two parameters were obtained: the guessing rate (g) and the standard deviation (sd) of the memory distribution. The reciprocal of the sd parameter represents the precision of visual working memory, and the calculation of the quantity K of visual working memory is given by: K= (1 - g) × N, where N is the size of the stimulus set. Additionally, in this study, the absolute deviation between participants’ responses on the color wheel and the actual values of the probed items was referred to as the offset, where a larger offset indicated a poorer performance in the visual working memory task. In this experiment, participants with a guessing rate greater than 0.75 were excluded from the data analysis.

The data were analyzed using SPSS 27.0. Considering the importance of the gender factor (Fatima et al., 2016; Jonasdottir et al., 2020), we first analyzed the comparison of gender and clinical variables between different groups using the chi-square test for categorical variables and the independent samples t-test for continuous variables. Then the experimental variables were subjected to two-factor repeated measure analysis of variance (ANOVA). The analyzed factors included the within-subject factor of delay duration (1 s, 4 s, and 6 s) and the between-subject factor of sleep quality group (high and low sleep quality). We used the Greenhouse–Geisser method to correct *p* values that did not conform to the spherical hypothesis, and we calculated eta-squared(η^2^_*p*_) as a measure of the effect size. The quantity and precision of visual working memory were analyzed in the experiment. To exclude the influence of fatigue effects on this experiment, the differences in the offset between the first 30 trials and the last 30 trials under all conditions were also analyzed across the two groups of participants.

## 3 Results

**Figure 2:**
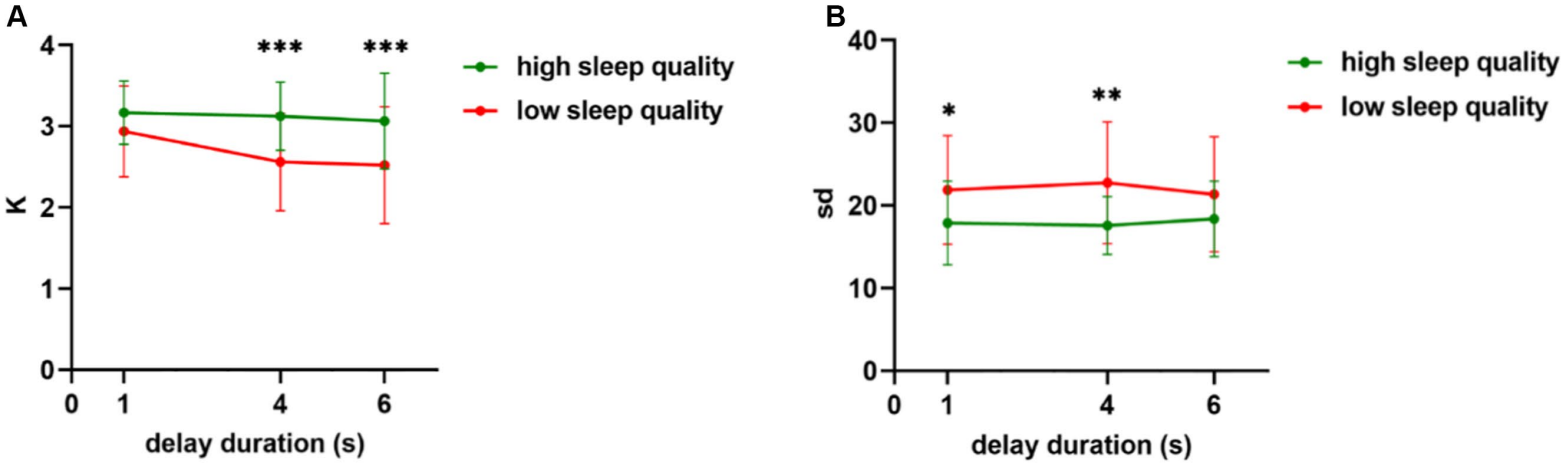
The results of the experiment. **(A)** Quantity (K) and **(B)** precision ^−1^ (sd) of visual working memory under high and low sleep quality conditions with a delay duration of 1 s, 4 s, or 6 s. The error target in the figure is the standard error; ^*^p < 0.05, ^**^p < 0.01, ^***^p < 0.001.

The results of the independent samples t-test for the gender factor showed that there was no significant difference in sleep quality between the female and male subjects, *t* (58) = 0.46, *p* = 0.65, Cohen’s *d* = 0.12. The results of two-factor repeated measurement ANOVA for variables are shown in Figure 2. As shown in Figure 2A, for the quantity (K) of visual working memory, the results indicated significant main effects of sleep quality (*F* (1, 58) = 8.97, *p* < 0.001, η^2^_*p*_ = 0.20) and delay duration (*F* (2, 57) = 6.44, *p* = 0.003, η^2^_*p*_ = 0.18). The interaction between sleep quality and delay duration was also significant (*F* (2, 57) = 3.87, *p* = 0.03, η^2^_*p*_ = 0.12). Further simple effect analysis revealed that there were no significant differences in visual working memory quantity between the high sleep quality group at 1 s and 4 s (*t* = 0.56, *p* = 0.58), 1 s and 6 s (*t* = 0.88, *p* = 0.52), and 4 s and 6 s (*t* = 0.66, *p* = 0.38) delay conditions. However, the low sleep quality group showed no significant differences (*t* = 0.42, *p* = 0.68) in working memory quantity between the 4 s and 6 s delay conditions, whereas both conditions showed significant differences compared to the 1 s delay duration. Specifically, the working memory quantity was significantly higher under the 1 s delay duration (M ± SD = 2.94 ± 0.56) than under the 4 s delay duration (M ± SD = 2.56 ± 0.60) (*t* = 4.38, Cohen’s *d* = 0.80, *p* < 0.001), and the quantity was significantly higher under the 1 s delay duration (M ± SD = 2.94 ± 0.56) than under the 6 s delay duration (M ± SD = 2.52 ± 0.72) (*t* = 3.07, Cohen’s *d* = 0.56, *p* = 0.005). For the 1 s delay condition, a marginally significant difference was evident between the high sleep quality group and the low sleep quality group (*p* = 0.07). By contrast, for the 4 s and 6 s delay conditions, significant differences in visual working memory quantity were apparent between the two groups (*p* < 0.01). These differences highlighted that more memory representations were retained by the participants in the high sleep quality group than in the low sleep quality group.

In terms of the precision^−1^ (sd) of visual working memory, the main effect of sleep quality was significant (*F* (1, 46) = 13.83, *p* < 0.001, η^2^_*p*_ = 0.19), whereas the main effect of delay duration was not statistically significant (*F* (2, 116) = 0.06, *p* = 0.94). The interaction between the two variables was also not statistically significant (*F* (2, 116) = 0.74, *p* = 0.48). Further simple effect analysis showed that the VWM precision of the high sleep quality group was significantly higher than that of the low sleep quality group at 1 s (*t* = 2.63, Cohen’s *d* = 0.68, *p* = 0.01) and 4 s (*t* = 3.47, Cohen’s *d* = 0.90, *p* = 0.001) delay conditions. See Figure 2B for details.

**Figure 3:**
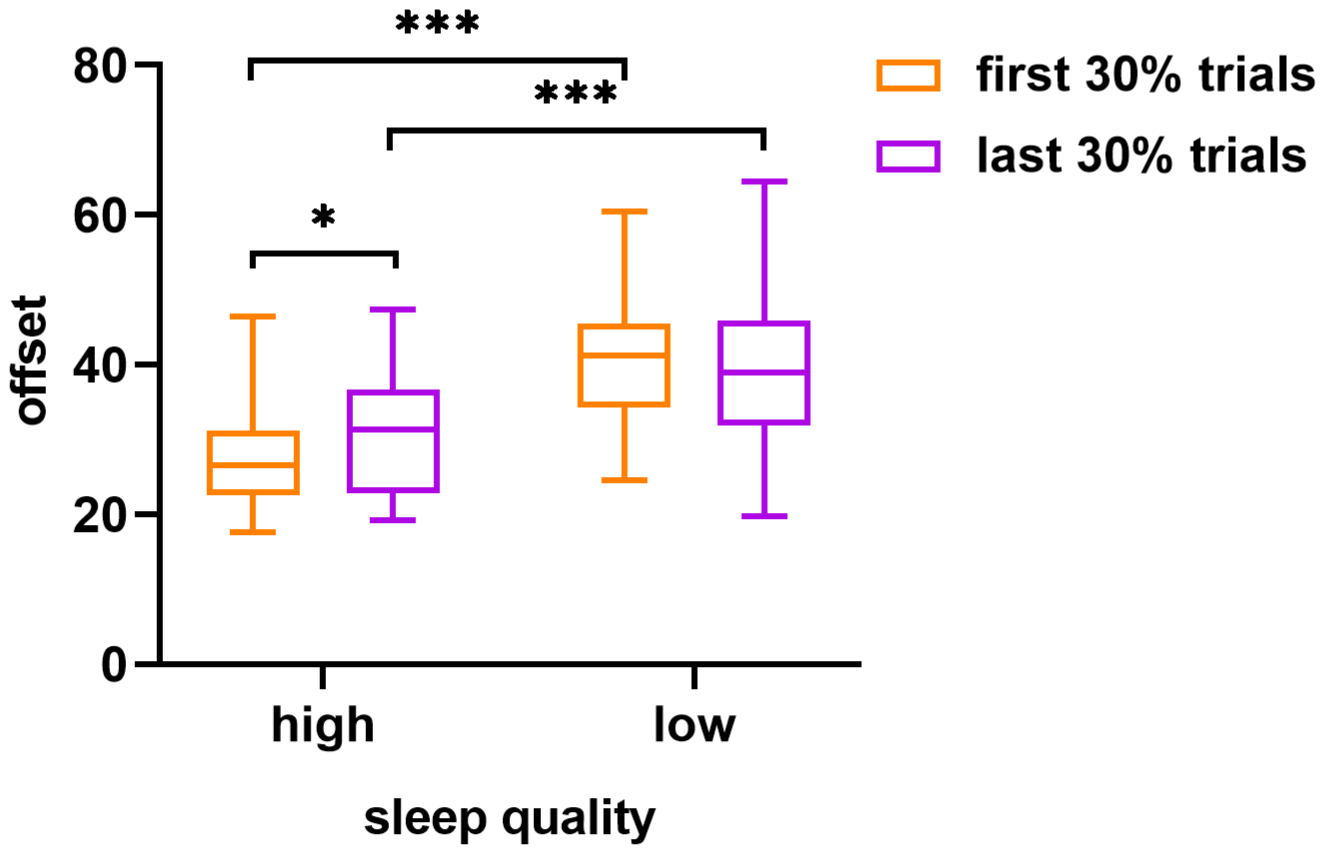
The difference in visual working memory task offset between the high and low sleep quality groups in different experimental orders (first 30% of the trials and last 30% of the trials). The error target in the figure is the standard error; ^*^p < 0.05, ^***^p < 0.001.

As shown in Figure 3, in terms of the offset, the main effect of sleep quality was statistically significant (*F* (1, 58) = 26.44, *p* < 0.001, η^2^_*p*_ = 0.31), whereas the main effect of the experimental order was not significant (*F* (1, 58) = 0.64, *p* = 0.43, η^2^_*p*_ = 0.01). The interaction between sleep quality and experimental order was significant (F (1, 58) = 4.66, *p* = 0.03, η^2^_*p*_ = 0.07). Further simple effects analysis revealed that the high sleep quality group showed significant differences in the offset of the visual working memory task under different experimental order conditions, whereas the low sleep quality group did not. Specifically, in the high sleep quality group, the offset was significantly lower for the first 30% trials (M ± SD = 27.86 ± 7.44) than for the last 30% trials (M ± SD = 30.72 ± 7.78), (*t* = 2.07, Cohen’s *d* = 0.38, *p* = 0.04). Within the first 30% of the trials, a significant difference was evident between the offset of the high sleep quality group (M ± SD = 27.86 ± 7.44) and the low sleep quality group (M ± SD = 40.63 ± 8.97), (*t* = 6.00, Cohen’s *d* = 1.55, *p* < 0.001). Similarly, within the last 30% of the trials, a significant difference was apparent between the offset of the high sleep quality group (M ± SD = 30.72 ± 7.78) and the low sleep quality group (M ± SD = 39.32 ± 10.90), (*t* = 3.52, Cohen’s *d* = 0.91, *p* < 0.001).

## 4 Discussion

The aim of this study was to investigate the impact of individual sleep quality on the maintenance phase of visual working memory by manipulating the maintenance duration of visual working memory information across three delay conditions (1 s, 4 s, and 6 s). Comparison of the behavioral performance of participants from the high and low sleep quality groups revealed several important findings. These provide new light on the role of sleep in visual working memory performance.

The results of this study indicate that that sleep quality is a significant predictor of individual visual working memory performance, as evidenced by various indicators, such as quantity, precision, and offset. On one hand, according to the alertness hypothesis, poor sleep quality leads to a decrease in alertness or physiological arousal in individuals, which in turn affects cognitive function (Lo et al., 2016; Hudson et al., 2020). Specifically, the hypothesis holds that sustained attention is a cognitive process that persists and is susceptible to sleep restriction and that the performance of working memory tasks is directly determined by sustained attention (Lim and Dinges, 2008). In other words, good performance can only be achieved if a certain level of alertness is also achieved and if attention is maintained on the task for a certain time. However, sustained attention is impaired when sleep quality deteriorates, resulting in interference with the visual working memory task. In the present study, a substantial investment of attention was required during the visual working memory maintenance phase (Chun, 2011; Long, 2023); consequently, the individuals with poor sleep quality had no capacity to keep their attention focused on the task at all times (Chua et al., 2017) and therefore showed poorer visual working memory performance. This suggests that the observed decline in visual working memory performance among individuals with poor sleep quality may be attributed to their difficulty in maintaining sustained attention on the task.

On the other hand, the influence of sleep quality on the maintenance of visual working memory may be closely associated with the enhancement and preservation of synaptic efficacy between neurons (Tononi and Cirelli, 2006, 2014; Feld and Born, 2020). In complex forms of movement, synaptic dynamic stability may be achieved through frequent use and non-utilitarian activation at rest. As the complexity of behavioral and perceptual activities increases, an accompanying rise also occurs in the demand for the storage and maintenance of memory information. These changes create conflicts between sensory input during wakefulness and synaptic homeostasis. However, adequate sleep can more effectively inhibit the processing of sensory input information, and particularly visual information, in the brain’s central processing area. Moreover, it can mitigate the conflict between information registration and dynamic stability, thereby maintaining memory (Kavanau, 1997; Caverzasio et al., 2018).

Extending the delay duration of information maintenance from 1 s to 4 s or 6 s creates a situation in which individuals with low sleep quality experience even greater challenges when attempting to sustain attention in visual working memory, particularly in terms of the quantity measure. This result is consistent with previous research (Xie et al., 2019). One possible reason for this result is that the maintenance of working memory information requires continuous investment of cognitive resources, while cognitive resources are already limited in people with poor sleep quality (Mauss et al., 2013; Kusztor et al., 2019). On this basis, if the delay time of the maintenance phase is extended (equivalent to increasing task difficulty), it will worsen the task performance of individuals with low sleep quality. Furthermore, at the neural level, sleep problems affect the function of the prefrontal and parietal cortex in individuals (Chee and Chuah, 2007; Tossell et al., 2023). And the frontal–parietal region is the main brain area responsible for the quantitative representation of visual working memory. This means that poor sleep will affect their visual working memory quantity representation by impairing the functional activity of the relevant brain regions. However, in terms of precision measures, no continuous decline was observed in task performance with increasing maintenance duration. This finding is consistent with the results reported by Zhang and Luck (2009), who found that individual visual working memory precision remained relatively stable as the maintenance duration increased. One possible explanation is that forgetting information in working memory may be an all-or-nothing process rather than a gradual decline process (Durstewitz et al., 2000; Zhang and Luck, 2009).

The findings of the present study also indicated a significant difference in the offset measure between the first 30% and last 30% of the trials in the high sleep quality group across all maintenance duration conditions. Specifically, the participants in the high sleep quality group showed a significantly lower offset measure in the first 30% of the trials than in the last 30% of the trials. This finding can be understood within the framework of the theory of attentional resources (Kahneman, 1973), which posits that individuals possess limited attentional resources. Task demands that exceed the available resources greatly impair an individual’s task performance (Cohen et al., 2012; Yao et al., 2022). As the number of trials increases across different experimental orders, the attentional resources available to individuals gradually decrease, creating challenges in sustaining sufficient attention during the maintenance phase of the visual working memory task. Consequently, performance tends to deteriorate in later trials.

Interestingly, the low sleep quality group did not exhibit the same change in performance between the early and later trials. One possible reason is that the performance of the low sleep quality group had already reached a floor effect, where the attentional resources had already decreased to their minimums at the start of the trials due to poor sleep quality. As a result, individuals with low sleep quality performed poorly right from the beginning of the experiment, and this state persisted throughout the experiment.

Through the above results, the present study found that sleep quality has a significant effect on the visual working memory maintenance stage. According to previous studies, it is known that sustained attention is more closely related to sleep quality and visual working memory (Kusztor et al., 2019; Olivers and Roelfsema, 2020; Mai et al., 2022; Liu et al., 2023; Li et al., 2024). However, whether the effect of sleep quality on the ability to maintain memorized information is dependent on the allocation and modulation of attentional resources needs to be further explored. Therefore, future studies could incorporate factors such as attention, mood, and an individual’s BMI to explore possible intermediate mechanisms between sleep quality and visual working memory. In addition, the future research can also combine sleep polysomnography, EEG and other devices, more objective and systematic to explore the impact of sleep quality on visual working memory processing. The mechanism between sleep quality and visual working memory can be explored more deeply in the future around these questions.

## 5 Conclusion

This study demonstrated that the effect of sleep quality on visual working memory changes with delay time. The longer the delay in the maintenance stage, the greater the cognitive demands individuals would have in completing the task. Consequently, individuals with low sleep quality performed worse in the visual working memory maintenance stage.

## Data availability statement

The data that support the findings of this study are available in the Figshare repository: https://doi.org/10.6084/m9.figshare.25304716.v1.

## Ethics statement

The studies involving humans were approved by the study was conducted in accordance with the principles outlined in the Helsinki Declaration, and approval was granted by the Ethics Committee of Liaoning Normal University (Approval number: LL2022032). The studies were conducted in accordance with the local legislation and institutional requirements. The participants provided their written informed consent to participate in this study.

## Author contributions

LG: Data curation, Formal analysis, Investigation, Methodology, Validation, Visualization, Writing – original draft, Writing – review & editing. MW: Data curation, Validation, Writing – original draft. CY: Methodology, Validation, Writing – original draft. QL: Conceptualization, Funding acquisition, Methodology, Project administration, Resources, Supervision, Writing – review & editing.
